# Is diabetic striatopathy the culprit of seizures in a patient with ketotic hyperglycemia-induced hemichorea–hemiballismus?

**DOI:** 10.1186/s12883-022-02659-5

**Published:** 2022-04-08

**Authors:** Abeer Sabry Safan, Omna Sharma, Muna Almasri, Ashton Ian D’Souza, Omer Suliman

**Affiliations:** 1 Department of Neurology, Neurosciences Institute, Hamad Medical Corporation, Doha, Qatar; 2 Weill Cornell Medicine-Qatar of Cornell University (WCM-Q), Doha, Qatar

**Keywords:** Diabetic striatopathy, Hemichorea-hemiballismus, Focal seizures, Generalized tonic-clonic seizures, Ketotic hyperglycemia, Case report

## Abstract

**Background:**

Diabetic striatopathy is a rare neurological manifestation of nonketotic hyperglycemia that presents with contralateral hemichorea-hemiballismus. Presentation with concurrent seizures is rarely reported.

**Clinical presentation:**

We report a case of diabetic striatopathy presenting with focal and generalized tonic-clonic seizures (GTCS) with right hemichorea-hemiballismus induced by a ketotic hyperglycemic state. Head MRI showed high T1-weighted signal intensity in the left lentiform nucleus with no significant diffusion restriction or postcontrast enhancement. The patient’s condition gradually improved, with seizure control on AEDs. Hemichorea-hemiballismus significantly improved with adequate blood sugar control and resolved with low-dose haloperidol.

**Conclusions:**

Diabetic striatopathy presenting with hemichorea-hemiballismus and concurrent GTCS has been reported previously in two cases; however, it has never been reported in ketotic hyperglycemia. To the best of our knowledge, we herein report the first case report of focal and generalized seizures in a ketotic hyperglycemic state and mesial temporal sclerosis.

## Background

Diabetic striatopathy (DS) is a rare movement disorder syndrome known as nonketotic hyperglycemia associated with hemichorea-hemiballismus (NKHCHB). It is associated with basal ganglia involvement manifesting with contralateral involuntary, continuous, nonrhythmic proximal dancing-like movements with a flailing component of one side of the body. It has an estimated prevalence of 1 in 100,000 patients. This syndrome has been previously reported in elderly patients with concurrent seizures in a nonketotic hyperglycemic state with complete or partial resolution of hemichorea-hemiballismus following glycemic control and serum osmolality.

We herein report a rare and first case of hemichorea-hemiballismus with concurrent focal and generalized seizures in a middle-aged male with ketotic hyperglycemia.

## Case presentation

A 45-year-old right-handed Nepalese male, nonsmoker, with a past medical history of noninsulin-dependent type 2 diabetes mellitus on oral hypoglycemic agents (OHA), nonadherent, brought to the accident and emergency (AE) department with first-time witnessed generalized tonic-clonic seizures and a two-week history of recurrent uncontrolled right-sided abnormal movements.

His right-sided abnormal movements were described as involuntary, nonpatterned, and dancing-like. They were initially intermittent but progressed to being continuous with intact awareness that interfered with activities of daily living (ADLs). Two days prior to hospitalization, he complained of holocranial tension-like headaches associated with nausea, dizziness, recurrent vomiting, abdominal cramps, and diffuse body ache, and the witnessed first-time generalized tonic-clonic seizures were aborted by 2 mg of intravenous (IV) lorazepam. There was no history of neck pain/stiffness, fever, preceding illness, or trauma, but he had an unintentional weight loss of eight kilograms over the past 2 months. A review of systems revealed no extraneurological manifestations or altered behavior. He did not have a significant family history of seizures or other movement disorders. Finally, he denied recent travel, sick contacts, or recent vaccination.

He developed recurrent brief (30–60 s) episodes of generalized tonic-clonic seizures, with consciousness regained in between, that required a loading dose of levetiracetam IV 1.5 g (gm) (nonstatus epilepticus dose) followed by a maintenance dose of levetiracetam 500 mg twice a day (BID). In subsequent days, with fluctuations in his blood glucose levels, he was witnessed to have focal seizure semiology with impaired awareness described as right head deviation with right forced gaze deviation and recurrent jerky movements of the right upper limb for 10 s followed by rapid regaining of consciousness. These focal seizures occurred at a frequency of up to 5 times/hour, warranting escalation of his levetiracetam dose to 1 g BID and reinitiating insulin infusion; these interventions controlled these focal seizures.

Initial vitals were within normal parameters: temperature of 36.4 °C, respiratory rate of 17 breaths per minute, blood pressure of 129/62 mmHg, and oxygen saturation of 98% on room air. Generally, he appeared emaciated and pale, with apparent abnormal and involuntary right-sided movements in a flailing and dancing pattern, suggestive of hemiballismus and hemichorea involving the face, trunk, arm, and leg. Cranial nerve examination was unremarkable, and he demonstrated normal symmetrical tone and power with upgoing plantar reflexes bilaterally, which were initially attributed to a postictal state in the absence of other pyramidal signs and its resolution upon discharge home. His gait was normal, with no cerebellar signs. The neuropsychological assessment was unrevealing, with a normal Mini-Mental State Examination (MMSE) score of 28/30.

Initial biochemistry workup revealed a serum blood glucose of 32.5 mmol/L, high serum ketones with beta-hydroxybutyrate levels of 5.81 mmol/L (normal range: 0.03–0.3), and urine ketone (+++) with normal venous pH of 7.39 and bicarbonate of 21. These findings suggested a ketotic hyperglycemic state [nondiabetic ketoacidosis (DKA)]. The DKA protocol was initiated with strict blood glucose monitoring, intravenous (IV) normal saline, and insulin infusion.

**Fig. 1:**
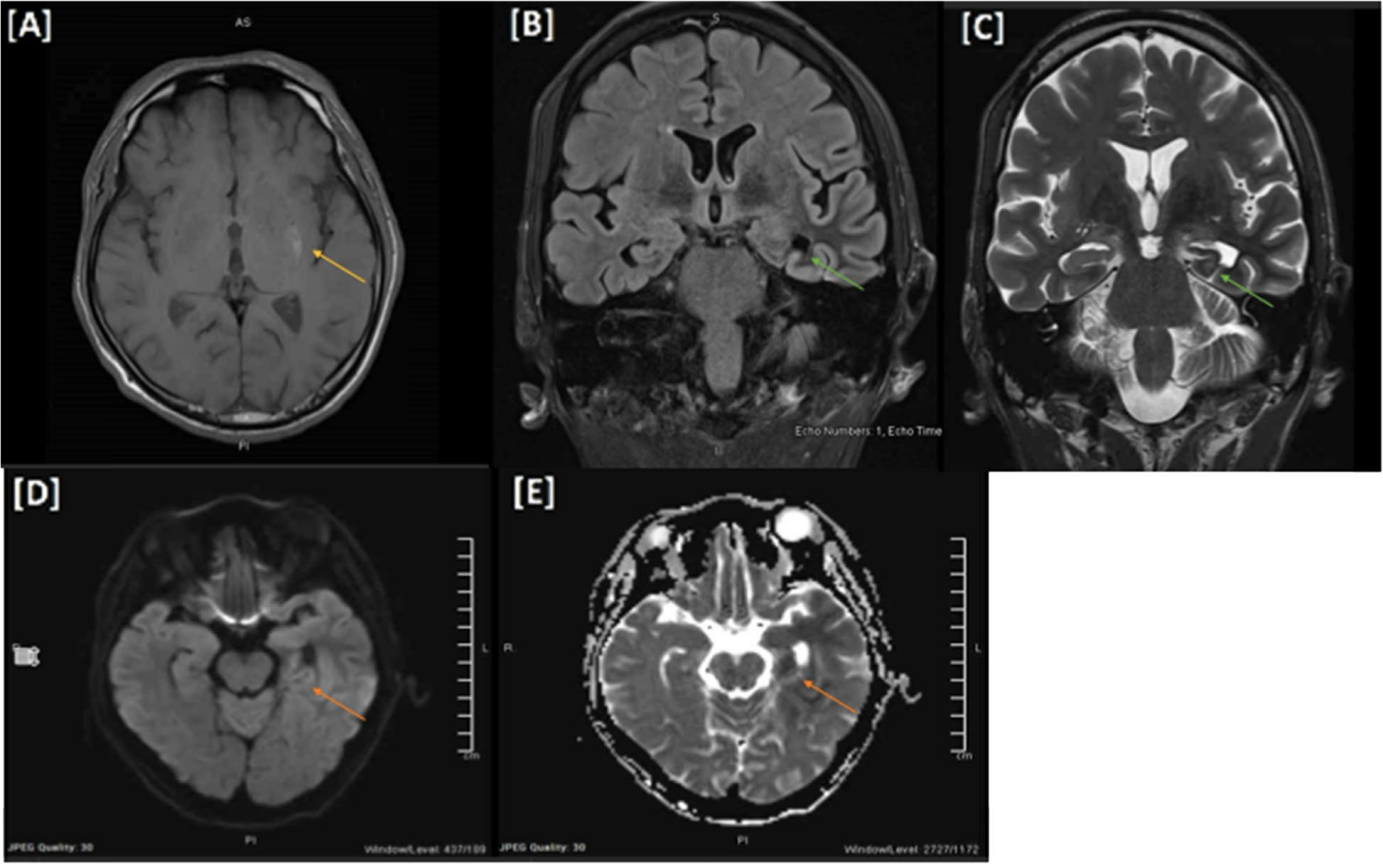
**A** High T1 signal intensity in the left lentiform nucleus with no significant diffusion restriction or postcontrast enhancement. **B**, **C** There are significant atrophic changes of the left hippocampus and left fornix with a subtle increase in signal intensity in FLAIR seen predominantly at the body, loss of interdigitation, and internal architecture bilaterally representing mesial temporal sclerosis. **D**, **E** Diffuse diffusion restriction, bright signal intensity on T2/FLAIR with faint postcontrast enhancement involving the left hippocampus and left temporal lobe cortex extending to the left insular and adjacent frontal cortices, likely representing postictal changes

Blood workup, including blood cell counts, thyroid function tests, and vitamin B12 levels, was within normal limits, with normal inflammatory markers. The autoimmune screening was unremarkable. Wilson’s disease workup revealed normal liver function tests (LFTs), ceruloplasmin and serum copper levels. Interictal (45 min) electroencephalogram (EEG) was initially obscured by muscle artifacts of the hemichorea-hemiballismus; however, it was repeated once movements and seizures were pharmacologically controlled with antiseizure medications. The EEG demonstrated alpha activity with no focal, diffuse, generalized abnormalities or epileptiform discharges. The head computed tomography (CT) scan was unremarkable. Head magnetic resonance imaging (MRI) with contrast revealed a hyperintense T1 signal in the left lentiform nucleus with no contrast enhancement (Fig. 1A), and left mesial temporal sclerosis (MTS) with left frontotemporal postictal changes on FLAIR with corresponding diffusion restriction that likely represented postictal changes (Fig. 1B-E). Susceptibility-weighted imaging (SWI) showed no blooming that suggested microhemorrhages. Lumbar puncture showed elevated CSF protein levels of 1.05 and elevated glucose levels of 6.81, with otherwise normal cytology, viral panel, and cultures. His high CSF protein was attributed to an acute severe hyperglycemic state, and he was treated with a working diagnosis of diabetic striatopathy with right hemichorea-hemiballismus induced by a ketotic hyperglycemic state.

The patient’s management encompassed a multidisciplinary approach with the addition of sodium valproate 500 mg BID and clobazam 1 mg BID, which resulted in seizure control. Additionally, blood sugar control and counseling were provided by a diabetic educator. On the fifth day after admission, upon normalization of serum glucose values (average between 80 and 120 mg/dL) and abolishing ketones, he was switched to a basal subcutaneous insulin regimen (insulin glargine 20 IU daily) and oral sitagliptin/metformin 50/500 mg BID. He demonstrated significant improvements in his right-sided hemichorea-hemiballismus throughout his hospital stay, with residual movements that were suppressed during sleep. After remaining seizure-free for 48 h on the 10th day of admission, clobazam was tapered down, and he was discharged home with a blood glucose diary, AEDs (sodium valproate 500 mg BID and levetiracetam 1 g BID), and an endocrine team follow-up.

At his six-week neurology outpatient follow-up visit, the patient was found to be doing well without seizure recurrence and adequate blood sugar control. Furthermore, his right hemichorea-hemiballismus was still present, impairing his activities of daily living; hence, he was started on haloperidol 0.5 mg BID, which resulted in almost complete resolution of the movements upon regular four-week follow-up. His hemichorea would return when distracted but was otherwise essentially absent at rest. Overall, 10 weeks following his initial admission, significant improvement and resolution of his hemichorea-hemiballismus symptoms and seizures were noted.

## Discussion and conclusion

Hemichorea-hemiballismus is a rare neurological manifestation observed in patients with poorly controlled diabetes in the context of hyperglycemic nonketotic crises that was first described in the 1960s [1]. It constitutes the triad of radiological striatal hyperintensity on T1-weighted MRI and contralateral hemichorea–hemiballismus with rapid resolution of the symptoms after glycemic normalization; hence, it is also labeled diabetic striatopathy [2]. Nonetheless, these abnormal movements have been described with other etiologies, such as subthalamic/basal ganglia cerebrovascular insults and autoimmune, toxic, malignant, and infectious illnesses [3].

The pathophysiology of diabetic striatopathy is mainly unknown; however, various theorized metabolic and pathologic mechanisms have been described in the literature based on diagnostic imaging, biopsies, and postmortem studies. In the state of nonketotic hyperglycemia, a metabolic shift toward anaerobic pathways is thought to cause inactivation of the tricarboxylic acid cycle, forcing cerebral metabolism into a GABA shunt [4]. GABA is utilized as an alternate energy source to produce succinic acid; however, this pathway only provides 10–40% of the energy required by the basal ganglia. In turn, this leads to a state of metabolic acidosis and subsequent basal ganglia dysfunction, clinically presenting as chorea [4]. The depletion of GABA, cellular acidosis, and regional metabolic decrease are believed to be the culprits behind the persistent abnormal movements 24–48 h after normalization of blood sugar [5]. However, this theory fails to explain the presence of chorea in the state of ketotic hyperglycemia, which the patient in this case report demonstrated.

A separate theory of diabetic striatopathy pathogenesis suggests it to be a sequela of cerebrovascular ischemia resulting from poorly controlled diabetes mellitus [4]. The state of hyperglycemia leads to structural destruction, as evidenced by vasculopathy and gliosis restricted explicitly to the striatum [2, 6]. Although evidence is limited, postmortem pathology and biopsy results of diabetic striatopathy have demonstrated neuronal loss, gliosis, and reactive astrocytosis within the basal ganglia [2]. The suggested pathophysiology involves neuronal injury and edema due to patchy ischemic injury, followed by parenchymal invasion of foamy macrophages and microglial activation [2, 6]. A lactate peak supports this theory, with regional metabolic failure supported by reduced glucose metabolism on PET in the areas corresponding to T1 hyperintensity on MRI [2]. Ischemic changes causing acute neuronal injury lead to the appearance of gemistocytes (swollen reactive astrocytes). Furthermore, it results in astrocytic hypertrophy potentially out of proportion to the degree of injury with low extracellular potassium and hyperpolarization leading to excessive neuronal discharge, which is epileptiform [7]. This theory is supported by proton MR spectroscopy, which revealed a decreased N-acetyl aspartate/creatine ratio in the affected basal ganglia and an increased choline/creatine ratio, which supports the presence of gliosis and decreased neuronal integrity [2].

Diabetic striatopathy more commonly occurs in elderly female patients of Asian descent with chronic and inadequately controlled diabetes mellitus (DM), which usually manifests with hemichorea-hemiballismus [2]. However, it is unclear whether a similar demographic pattern exists in ketotic hyperglycemic seizures. Sato et al. reported a case of DS with altered sensorium in the absence of any extrapyramidal signs (hemichorea-ballismus), suggesting possible variability in the clinical presentation of striatal lesions [8]. Chua et al. recently published a review article on 176 patients with diabetic striatopathy, which estimated that 96.6% had type II DM and 3.4% had type 1 DM; the average blood glucose level was 23 mmol/L with 13.1% glycated hemoglobin (HbA1c) [3]. With striatal region involvement, corresponding hemichorea-hemiballismus commonly affected the arm-leg (58.6%) and arm-leg-face (22.1%, 32/145), with the least common clinically observed involvement being arm-leg-face-trunk (0.7%, 1/145) [3]. However, bilateral right facial-arm-leg involvement was observed in 9.6% of reported DS [3]. Our patient was middle-aged with an HbA1c of 16%, falling into the rare subgroup of exclusive right-sided involvement. Radiologically speaking, magnetic resonance imaging (MRI) studies have revealed that diabetic striatopathy most commonly affected the putamen (94.1%, 144/153), as seen in our patient, followed by the caudate nucleus (64/153) and globus pallidus (43/153); however, bilateral striatal involvement has been reported in 9.7% of DS patients [3].

There are two types of hyperglycemia—nonketotic and ketotic. Traditionally, seizures in hyperglycemia are focal rather than generalized and are more common in nonketotic states [9]. While there have been cases of seizures in nonketotic hyperglycemia (NKH) reported in the literature, none have been reported for ketotic hyperglycemia, which could be due to the protective effect of ketones against seizures [10]. Moien-Afshari et al. described a case in an elderly gentleman with a history of noninsulin-dependent DM and ischemic heart disease who presented with episodic jerking of his left upper limb accompanied by visual symptoms of flashing lights in the ipsilateral visual field [11]. They described symptom resolution of his focal occipital seizures within 24 h with insulin administration and adequate hydration. Likewise, Chung et al. and Kim et al. described cases where patients had NKH and developed hemichorea and focal seizures with temporo-occipital lobe involvement [12, 13]. Our patient had semiology of new-onset focal seizures with secondary generalization in the context of ketotic hyperglycemia and a left MTS. While we believe MTS predisposed him to seizures, both left diabetic striatopathy and MTS are possible epileptogenic zones, as focal motor seizures have been well described previously in NKHG [14].

DS is mainly treated by correcting the underlying metabolic disturbances via glucose control and adequate hydration [15]. According to a systematic review by Chua et al., only a quarter of patients with DS demonstrated a complete response to glucose control alone, and 76.2% of patients needed the addition of antichorea medications for successful treatment [14]. These medications include antipsychotics, selective serotonin reuptake inhibitors, GABA receptor agonists, and dopamine-depleting agents [15]. These drugs may be used alone or in combination. Other approaches, such as transcranial magnetic stimulation and globus pallidus internus deep brain stimulation, can be used for symptom control in patients with chorea refractory to glucose control and medication. The review reported that the use of antichorea medications showed a high degree of effectiveness for chorea relief in patients unresponsive to strict glucose control. Haloperidol was the most common medication used for treatment in DS and proved to be effective in most cases. Regardless of the treatment, the overall recurrence rate of chorea was 18.2%. Therefore, regular follow-ups are needed after the resolution of symptoms [3]. In our case, the patient’s hemichorea-hemiballismus improved dramatically with strict blood glucose control and low-dose haloperidol, with a plan to taper haloperidol over subsequent follow-up visits [1].

## Data Availability

The datasets used and/or analyzed during the current study are available from the corresponding author on request.

## Abbreviations

OHA: Oral hypoglycemic agents
DS: Diabetic striatopathy
NKHCHB: Nonketotic hyperglycemia associated with hemichorea-hemiballismus
NKH: Nonketotic hyperglycemia
GABA: Gamma-aminobutyric acid
ADLs: Activities of daily living
AED: Anti-epileptic drug
CSF: Cerebrospinal fluid
EEG: Electroencephalogram
ED: Emergency department
GTCS: Generalized tonic-clonic seizures
MTS: Mesial temporal sclerosis
MMSE: Mini-Mental State Examination
MRI: Magnetic resonance imaging
DTR: Deep tendon reflexes
LP: Lumbar puncture
FLAIR: Fluid attenuated inversion recovery
ANA: Autoimmune screen (anti-nuclear antibody)
ANCA: Antineutrophil cytoplasmic antibody
